# Extracellular Acidification Induces Lysosomal Dysregulation

**DOI:** 10.3390/cells10051188

**Published:** 2021-05-13

**Authors:** Bryce Ordway, Robert J. Gillies, Mehdi Damaghi

**Affiliations:** 1 Department of Cancer Physiology, H Lee Moffitt Cancer Center and Research Institute, Tampa, FL 33612, USA; Bryce.ordway@moffitt.org (B.O.); Robert.gillies@moffitt.org (R.J.G.); 2 Department of Oncological Sciences, University of South Florida, Tampa, FL 33612, USA

**Keywords:** cancer acidosis, lysosome dysregulation, lysosomal localization, tumor microenvironment, Warburg effect, targeted therapy, breast cancer, cancer metastasis, intracellular pH

## Abstract

Many invasive cancers emerge through a years-long process of somatic evolution, characterized by an accumulation of heritable genetic and epigenetic changes and the emergence of increasingly aggressive clonal populations. In solid tumors, such as breast ductal carcinoma, the extracellular environment for cells within the nascent tumor is harsh and imposes different types of stress on cells, such as hypoxia, nutrient deprivation, and cytokine inflammation. Acidosis is a constant stressor of most cancer cells due to its production through fermentation of glucose to lactic acid in hypoxic or normoxic regions (Warburg effect). Over a short period of time, acid stress can have a profound effect on the function of lysosomes within the cells exposed to this environment, and after long term exposure, lysosomal function of the cancer cells can become completely dysregulated. Whether this dysregulation is due to an epigenetic change or evolutionary selection has yet to be determined, but understanding the mechanisms behind this dysregulation could identify therapeutic opportunities.

## 1. Introduction

Unlike most epithelial tissue, cancer cells go through an energy production process called the Warburg effect. The Warburg effect describes the phenomena occurring when a cell upregulates fermentative glycolysis, despite the presence of oxygen in the environment. This process results in an increase in glucose consumption and an increase in lactic acid production by cancer cells [1]. Lactic acid produced during aerobic glycolysis is ultimately shuttled out of the cell by monocarboxylate transport (MCT) proteins, which leads to the acidification of the extracellular environment surrounding cancer cells [2]. The acidification of the extracellular environment surrounding breast cancer cells has a wide variety of effects, one of which being altered lysosomal trafficking. When exposed to an acidic extracellular environment, breast cancer cells were shown to have an increase in lysosomal displacement in relation to the cell nucleus [3]. Movement of lysosomes to the cell periphery will have effects on the signal transduction from lysosomal associated signaling complexes and luminal pH of the lysosome [4]. This movement of lysosomes to the cell periphery when exposed to an acidic extracellular pH (pHe) is not unique to cancer and has been characterized many times before in a variety of different cell types [5]. From an evolutionary perspective, if this lysosomal displacement is seen across many different cell types, including non-pathogenic cell types, it must present some survival benefit to cells exposed to low pH conditions. Peripheral lysosome distribution can have a variety of positive and negative impacts on cancer cell fitness and progression. One possible benefit is the resulting increased secretion of extracellular matrix (ECM) degrading enzymes, which will ultimately lead to a decrease in ECM density, increased mobility, and metastasis. If lysosomal displacement to the cell periphery is providing a survival and metastatic benefit to cancer cells in acidic environments, this may present a possible therapeutic target if this process is able to be disrupted.

## 2. Extracellular Acidification and Its’ Effect on Lysosome Trafficking

### 2.1. Molecular Mechanisms of Extracellular Acidification in Cancer

**Figure 1:**
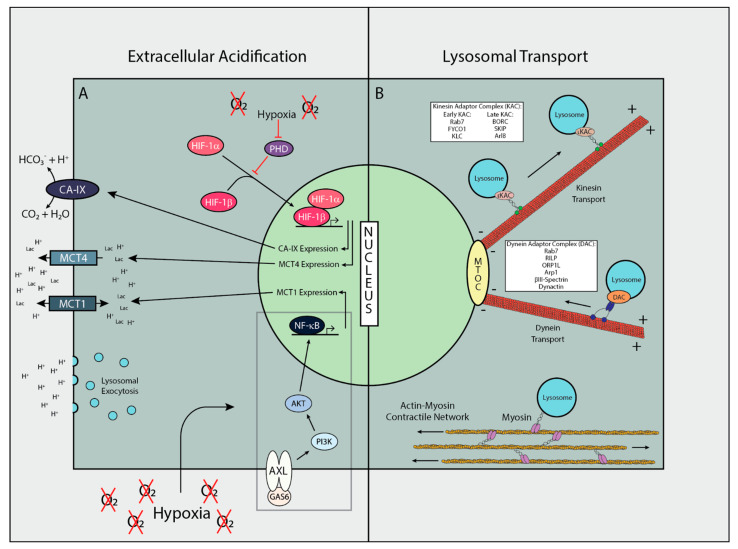
Mechanisms of Extracellular Acidification and Lysosomal Transport. (**A**) Extracellular milieu can be acidified by acid protrudes and pumps such as MCTs and CAs. Expression of these proteins that is influenced by external stimuli such as hypoxia changes the cells phenotype adapted to new microenvironment. (**B**) Main routes of lysosomal transport within the cell, and the proteins associated with each process. Dark grey—Cell body, Orange—Microtubules, Yellow—Actin Filaments, Light blue—Lysosomes. MTOC, Microtubule Organizing Center.

The reason why cancer cells have high aerobic glycolysis is still up for debate [6,7], but the main players that contribute to the acidosis are well characterized. Major players in the extracellular acidification in cancer are the MCT proteins, which are responsible for shunting lactate out from the cell leading to acidification of the extracellular environment. In order for this flux to be meaningful, MCT proteins, specifically MCT1/4, need to have an increase in expression in order to move the large amounts of accumulated lactate and H^+^ (Figure 1A). In cancer it has been previously shown that NF-κB is necessary for upregulation of MCT1 under hypoxic conditions, and outside the presence of p53 [8], although the function interaction between hypoxia and NF-κB was not determined. NF-κB activation must occur prior to its initiation of MCT1 expression, and can be caused by the AXL-Akt signaling cascade [9]. As hypoxia has also been shown to stabilize Gas6/AXL signaling [10], it is possible this closes the pathway from hypoxia to MCT1 upregulation (Figure 1A). This signaling process has been shown to promote the invasion and metastasis of cancer, with an emphasis on the receptor tyrosine kinase AXL as the main node in this pathway [11]. In contrast to MCT1, the expression of MCT4 is under direct control of a hypoxia response element (HRE) meaning that its expression is dependent on hypoxic conditions and the activation of HIF-1α. The bicarbonate buffering membrane proteins, Carbonic Anhydrases (CA), have also been heavily implicated in regulating the extracellular pH environment within solid tumors (Figure 1A). Carbonic Anhydrase 9 (CA-IX) has been shown to directly regulate the extracellular environment as a pH-stat in solid tumors, with tumors expressing CA9 having a pH 0.15 lower than control tumors [12]. As with MCT4, CA9 expression is dependent on the activation of HIF-1α, a testament to the reliance of extracellular acidification on the hypoxia phenotype, dependent or independent of oxygen concentrations. HIF-1α expression is under control of the transcription factor STAT3, which will be discussed in subsequent sections. It is also important to note that lysosomal exocytosis will also contribute to the extracellular acidosis due to the low pH of the lysosomal lumen that is created by V-ATPases [13,14].

### 2.2. Mechanisms of Lysosomal Transport

An important aspect of lysosomal functioning is the directed trafficking of lysosomes throughout the cell. Trafficking of lysosomes is carried out via motor proteins that bind to both lysosomes and cytoskeletal filaments in order to move the lysosomes in a desired direction. For microtubular movement the motor proteins Kinesin and Dynein are used. Kinesin generally moves lysosomes in a plus end direction, toward the cell periphery, whereas Dynein generally moves lysosomes in a minus end direction, toward the microtubule organizing center, MTOC. Actin-based lysosomal transport is mediated by Myosin motor proteins that can move lysosomes bidirectionally along actin. Each of these motor proteins also require a complex of multiple proteins to facilitate their binding and processing, which can allow regulation of lysosomal movement. The Kinesin adaptor complex (KAC) consists of the adaptor proteins, Rab7, FYCO1, and KLC, which swap with BORC, SKIP, and Arl8 during late stages of Kinesin processivity (Figure 1B). Upregulation of some of these adaptor molecules has been shown to increase Kinesin processivity and drive peripheral distribution of lysosomes [15]. The Dynein adaptor complex (DAC) consists of the proteins, Rab7, RILP, ORP1L, Arp1, βIII-spectrin, and Dynactin (Figure 1B). Conversely to Kinesin, upregulation of some of these adaptor molecules can cause lysosome accumulation around the MTOC. Unlike Kinesin and Dynein, the binding of Myosin to lysosomes is not as well studied and no adaptor molecules have been identified, but the transport of lysosomes by Myosin has been reported frequently. Due to the difference in structure between actin and microtubule networks, the movement of lysosomes via Myosin does not result in unidirectional transport to and from the cell center. Actin networks are generally more intertwined and web-like compared to microtubule networks, localizing around the plasma membrane. This means isolated lysosomal movement mediated by Myosin is less processive, but when taking into account the entire Actin­–Myosin network, lysosomes can be moved over long distance toward the cell periphery by contractions in the network as a whole [16] (Figure 1B).

## 3. Causes and Consequences of Acid Induced Lysosomal Dysregulation

### 3.1. Lysosomal Response to an Acidic Microenvironment

**Figure 2:**
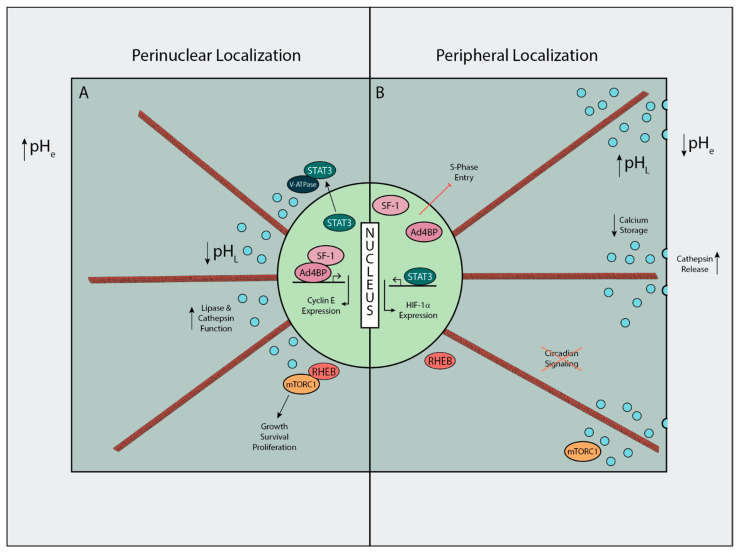
Lysosomal Localization and its’ Effects on Cellular Processes. (**A**) Signaling cascades and phenotype associated with a perinuclear lysosomal localization. (**B**) Signaling cascades and phenotypes associated with a peripheral lysosomal distribution. Dark grey—Cell body, Light blue—Lysosomes, Orange—Microtubules.

As mentioned previously, the net effect of an acidic microenvironment on lysosomes in cancer and normal epithelial cells is a redistribution from a peri-nuclear location to a more peripheral, sub-plasmalemmal location. Glunde et al. described a heterogeneous response to change in lysosomal diameter in response to extracellular acidosis, and a consistent drop in lysosomal number in their panel of breast cancer cell lines [3]. Previous studies have shown that the pH of lysosomes differs based on their position within the cell. Knocking out Arl8b induces a peripheral lysosome phenotype and it was found that the Arl8b knockout had an average lysosomal pH of nearly 6 compared to a control pH of 5.1 [4]. These peripheral lysosomes did not have an increase in interactions with the extracellular membrane based on colocalization analysis of immunofluorescent images. It has also been demonstrated that intracellular pH can influence the positioning of lysosomes [17], however, no study has shown that directly altering the pH of lysosomes can alter their positioning. STAT3 is an important regulator of intracellular pH dynamics. Under conditions of acidic intracellular pH, STAT3 is removed from its position as a transcription factor and associates with the lysosomal V-ATPase to move protons from the cytosol to the lysosomal lumen [18] (Figure 2A). With what is known about the reverse pHi effect in cancer cells [19] and the localization of lysosomes by luminal pH, this would provide a connection between the extracellular pH environment and peripheral lysosomal placement.

### 3.2. Consequences of Acid Induced Lysosomal Dysregulation

The positioning of lysosomes in relation to the nucleus has surprising effects on cell signaling with respect to number of pathways and function. Many years ago, it was shown that elevated levels of various lysosomal proteases, including some cathepsin family proteins, were present in the extracellular environment surrounding breast cancer cells, as highlighted in the early review by Rochefort et al. [20]. Implications for this increased extracellular cathepsin levels were initially not known, but it is now known that cathepsins are involved in degrading the ECM surrounding tumor cells, a hallmark of tumor invasion [21]. An early study with chloroquine, a drug known to neutralize the acidity of the lysosomal lumen, demonstrated that treatment with chloroquine causes an increased secretion of acid hydrolases [22], which we presume includes lysosome associated cathepsins. As mentioned previously, the luminal pH of lysosomes and their positioning are intertwined, pointing to the previously demonstrated phenomena of peripheral lysosomal distribution causing increased cathepsin release [23] (Figure 2B).

Many proteins have distinct cellular localization and some of these proteins rely on the position of lysosomes to determine their association with lysosomal signaling complexes. One example of this is the association between Ras homolog enriched in brain (RHEB) and mammalian/mechanistic target of Rapamycin complex 1 (mTORC1), the master regulator of cellular nutrient response. RHEB is localized to the perinuclear space and activates the mTORC1 complex when lysosomes are co-localized in the perinuclear space (Figure 2A). In order for this to happen, mTORC1 must be translocated to the lysosomal surface, which is driven by the amino acid sensing V-ATPase/Regulator complex [24]. Upon treatment with a low pH environment, the lysosomes move to periphery and decrease the associations made between RHEB and mTORC1 (Figure 2B). Ultimately, this leads to the inhibition of the circadian oscillations which are directed by mTORC1 signaling [25]. A review authored by Sulli et al. provides an overview of the current data on the effects of suspending the circadian clock in relation to cancer, from epidemiological and genetic studies to the molecular pathways of the circadian clock [26]. They propose an interplay between the CLOCK/BMAL1 and the cancer associated MYC and HIF-1α, in which suspension of the circadian clock could alter the oscillation of glycolysis. Suspension of the circadian clock by MYC has been shown to increase the glycolytic phenotype in cancer cell culture [27]. Peripheral positioning of lysosomes has been shown to affect the signaling of not only mTORC1, but also mTORC2, which were both shown to have quicker reactivation by growth factor stimulation when in peripheral cytoplasmic positioning [28].

Assuming that the luminal pH of lysosomes is dependent on the localization within the cell, the acid induced peripheral displacement of lysosomes would have effects on their functioning. Many of the lysosomal proteins that carry out recycling functions rely on the acidic pH of the lysosome to carry out their catalytic functions. The peripheral lysosomes have a pH near 6.0, making them significantly more alkaline than is optimal for lipases and cathepsins [29,30,31]. Overall, this would decrease the catabolic function of the lysosomes and lower their capacity to recycle macromolecules. Increasing the pH of lysosomes has also been shown to severely decrease levels of lysosomal calcium [32] (Figure 2B). Combining this knowledge with the fact that the endoplasmic reticulum (ER) is responsible for replenishing lysosomal calcium [33], it is possible that the decreased lysosomal calcium caused by increased lysosomal pH is due to their peripheral displacement caused by the pH, moving them away from the perinuclear ER. As a main calcium store within the cell, improper calcium regulation by lysosomes can have major effects and has been linked to a wide variety of pathological conditions deemed lysosomal storage disorders (LSDs) [34]. Lysosomal calcium signaling has also been implicated in cancer. A recent review by Wu et al. has summarized the role of lysosomal calcium channels in the regulation of autophagy, a metabolic pathway of increasing interest in cancer [35,36]. TRPML2, a lysosomal calcium channel, has been shown to increase the survival and proliferation of glioma cells when overexpressed [37]. TRPML2, and other TRPML family members, are responsible for exporting calcium from the lysosomal lumen [38]. The direct effect of TRMPL2 overexpression would be lower lysosomal calcium, as seen in peripheral lysosomal displacement. Much like TRMPL2, the lysosomal calcium channel Two-pore channel 2 (TPC2), has also been of increasing interest as a potential target and biomarker in cancer, such as melanoma, although the exact role it plays in the pathogenesis remains unknown [39].

When the cellular plasma membrane becomes damaged, it is the role of the lysosome to aid in the repair of said membrane; a process which has been shown to be facilitated by calcium (Ca^2+^) mediated exocytosis [40], followed by mass endocytosis [41]. This process is vitally important as to not let the cytosolic milieu spill into the extracellular environment, and maintain the structural integrity of the cell. The source of vesicles for the Ca^2+^ mediated exocytosis in non-secretory cells was later demonstrated to be proximal lysosomes [42]. It is now known that the luminal contents of the lysosome’s secreted into the extracellular environment are vital for carrying out membrane repair, as lysosomal proteases have been demonstrated to play a role during membrane repair [43], but it is still not totally clear if the Ca^2+^ used in the repair process is of extracellular or lysosomal origin. As all of these processes rely on the peripheral localization of lysosomes, it is possible that this repair mechanism could be enhanced by the acid induced redistribution of lysosomes.

Proper lysosomal regulation is also important for the maintenance of proper cell cycle dynamics. As mentioned previously, lysosome displacement can suspend the circadian clock. The circadian clock cycle is heavily intertwined with the cell cycle, and disruption of the circadian clock can have a variety of direct effects on cell cycle [44]. Inhibition of lysosomes by neutralizing their luminal pH has been shown to reduce the stability of the Ad4BP/SF-1 complex. Reduced stability of the Ad4BP-SF-1 complex reduces the entry to S-phase via alteration of Cyclin E1 expression [45] (Figure 2B). Recently, lysosomes have been shown to have an immediate role in controlling mitosis by releasing Cathepsin B (CTSB) during prometaphase, which localizes at chromatin during metaphase to cleave histone H3 [46]. Inhibition of this process leads to the formation of micronuclei, which have been heavily implicated as harbors of gene duplicates and markers of genetic instability, a hallmark of cancer [47,48,49]. More directly, the inhibition of vacuole/lysosome biogenesis has been shown to cause cell cycle arrest in early G1 phase due to a broken TORC1-SCH9 signaling pathway. This has been proposed as a possible checkpoint for intact vacuole/lysosome functioning [50].

### 3.3. Theoretical Benefits of Peripheral Lysosome Trafficking in Response to Acidic Tumor Microenvironment

In 1860, the French physiologist Claude Bernard noted in his Cahier Rouge that “The constancy of the internal milieu is a prerequisite of all living things” [51]. Hence, the plasma membrane is the ultimate structure to divide the internal cytoplasmic milieu from the harsh extracellular microenvironment. Exposure to an acidic extracellular environment has adverse effects on the plasma membrane and can damage the lipids that are generally present on the plasmalemmal outer leaflet. One way to reverse and prevent this damage would be to replace these acid sensitive lipids with lipids that are better suited for, or protected from, acid exposure. It has been shown previously that cells adapted to an acidic extracellular environment have increased expression of LAMP2b and increased representation on the plasma membrane [13,52,53,54]. Due to the acidic nature of the lysosomal lumen, the lipid composition of the inner leaflet of the lysosomal membrane has an increased concentration of lipids that are resistant to acid hydrolysis. One lipid that is exclusively located in the membrane of endosomal and lysosomal membranes is bismonoacylglycerophosphate (BMP) [55]. It has been shown that BMP has the ability to maintain a negative charge in the low pH environment of the lysosomal lumen [56], a testament to its buffering capacity. If a lysosome was to exocytose and fuse with the plasma membrane, the lipids present on the inner leaflet of the lysosomal membrane would now be present on the outer leaflet of the plasma membrane and protect the cell from the harsh acidic extracellular environment. This concept of lysosomal membrane components protecting the plasma membrane in acidic tumor microenvironments is not isolated to the lipids of lysosomes, and extends to the membrane proteins. LAMP proteins maintain a glycolic sugar coating on the inside of the lysosome that helps to protect the membrane from degradation. Moving these proteins to the outer leaflet of the plasma membrane would have significant benefits for the cell when exposed to the acidic tumor microenvironment, and this could be done via the exocytosis of lysosomes.

## 4. Targeting Lysosomes in Cancer Therapy

Modulation of the acidosis-lysosome signaling cascade has recently been of increasing interest. Many proteins involved in these pathways have been the target of small molecule drug design, or have had drugs targeting them repurposed for the objective of treating cancer (Figure 1). Among these, pH-sensing proteins are of heightened interest. Transient Receptor Potential Vanilloid1 (TRPV1) is a pH-sensing membrane protein that modulates the function of lysosomes via Ca^2+^ signaling. TRPV1 has been shown to be significantly upregulated in breast cancer [57], and both TRPV1 agonists [57] and antagonists [58] have been shown to have anti-cancer effects. Acid-sensing ion channel (ASIC) proteins also play a role in transferring extracellular pH into a lysosomal response, and have been targeted in the effort to treat cancer. Psalmotoxin 1 (PcTX1) is a blocker of the ASIC isoforms ASIC1 and ASIC2, which have been shown to promote the proliferative and migratory effects of extracellular acidosis on cancer cells. APETx2 is a peptide isolated from sea anemone and has been shown to have an inhibitory effect on ASIC3 [59]. ASIC3, along with ASIC1, has been implicated in inducing acid-mediated EMT in pancreatic cancer [60], and have been shown to be expressed in Glioblastoma [61]. Another peptide isolated from sea anemone, PhcrTx1, is also an acid-sensing ion channel inhibiting molecule that targets ASIC3 and ASIC4 [62]. In previous publications we have demonstrated the efficacy of targeting the Warburg phenotype, via inhibition of glycolysis, for controlling aggressive cancer populations [19,63]. As stabilization of Ad4BP/SF-1 has been shown to be necessary for glycolytic function, one could imagine that this may present a therapeutic opportunity by either targeting the complex directly, or inhibiting lysosomal functions. One of the most promising targets, palmitoyl-protein thioesterase 1 (PPT1), is a palmitoyl transferase that engages in the depalmitoylation of cathepsins prior to their degradation. PPT1 is an interesting lysosomal protein due to its’ high pH optimum of 7.0 [64], a pH closer to what is seen in peripheral lysosomes. PPT1 has been shown to elicit antitumor activity when targeted in models of melanoma and colon cancer [65,66]. GNS561, targeting PPT1 that has been shown to have significant antitumor activity against hepatocellular carcinoma in models is in current clinical trials. It has been shown to alter Zn^+^ accumulation, cathepsin activity, autophagic flux, and mTOR localization [67]. With all this being said, there is some concern about the targeting of lysosomes by therapeutics due to the diversity and importance of proper lysosomal function. However, targeting lysosomal proteins or membrane lipids that are exposed on the cell surface that is unique to cancer cells might be a great opportunity for drug development.

## 5. Conclusions

In total, we have presented the currently known causes and consequences of acid-induced lysosomal dysregulation. It is clear that this system is highly complex, and requires direct attention for its study and understanding. Our current understanding of the mechanisms behind the peripheral displacement of lysosomes when cells are exposed to an acidic environment is ill-defined and should be studied to focus our view of the system and possibly open a therapeutic window. The consequences of the lysosomal dysregulation we presented are highly relevant to cancer and may play vital roles in the initiation, survival, and progression of cancer. As presented, the targeting of lysosomal pathways in cancer is of great interest and shows promise in animal models of the diseases. Still, the widespread importance of lysosomal function in healthy tissue requires acknowledgment when investigating the toxicity of targeting these pathways.

## Data Availability

Not applicable.
